# Metropolitan Hospital Sunday Fund

**Published:** 1892-08-06

**Authors:** 


### The Institutional Workshop.

### METROPOLITAN HOSPITAL SUNDAY FUND.

The following is the report of the Committee of Distribution adopted by the Council on the 29th ult., the Right Hon. David Evans, Lord Mayor, in the chair :—

Your Committee have the honour to recommend the payment of awards to the 173 institutions enumerated below, being three less than last year, and an increase of 68 since the first awards were made in 1873. The total amount available for distribution, after allowing for liabilities and the usual current expenses, is £40,228. Of this total £40,118 15s. is now recommended to 120 hospitals and 53 dispensaries. Five per cent. of the total collected—viz., about £2,000—is set apart to purchase surgical appliances. Your Committee recommend that all payments to the Fund after August 1st be carried to the credit of next year's Fund.

In compliance with an order of the Council, and for the special use of its members, tables of statistics have been prepared as usual, showing an analysis of the number of beds in hospitals, the cost of patients both at hospitals and dispensaries, the proportionate expense of management, as well as other valuable information, and copies are sent to every member of the Council.

The number of deputations representing committees of various hospitals, &c., invited to confer with your Committee, and to offer explanations on matters of apparently unsatisfactory character, was sixteen. Of these, seven elected to leave their awards entirely in the hands of this Committee. Nine were invited to send deputations to meet your Committee ; of these, one is recommended for award on its full basis, five on a reduced scale, and three are not considered to be entitled to anything. Awards are therefore recommended to 157 institutions on their full bases, to thirteen institutions on reduced bases ; and three institutions which have made applications are not recommended for any award.

### PARTICULARS OF THE AWARDS FOR 1892.

#### Twenty-four General Hospitals.

Charing Cross Hospital, £1,093 15s. ; French Hospital, £291 13s. 4d. ; German Hospital, £625 ; Great Northern Central Hospital, £364 11s. 8d. ; Guy's Hospital, £625 ; Italian Hospital, £93 15s. ; King's College Hospital, £1,458 6s. 8d. ; London Hospital, £3,125 ; Metropolitan Hospital, £520 16s. 8d. ; Miller Hospital and Royal Kent Dispensary, £281 5s. ; North-West London Hospital, £312 10s. ; Poplar Hospital, £229 3s. 4d. ; Queen's Jubilee Hospital, £83 6s. 8d. ; Royal Free Hospital, £937 10s. ; St. George's Hospital, £1,354 3s. 4d. ; SS. John and Elizabeth Hospital, £125 ; St. Mary's Hospital, £2,187 10s. ; Seamen's Hospital Society, £854 3s. 4d. ; the Middlesex Hospital, £1,979 3s. 4d. ; Training Hospital, Tottenham, £312 10s. ; University College Hospital, £1,302 1s. 8d. ; West London Hospital, £604 3s. 4d. ; Westminster Hospital, £1,093 15s. ; London Temperance Hospital, £541 13s. 4d.

#### Special Hospitals.

Five Chest Hospitals.—City of London Hospital for Diseases of the Chest, Victoria Park, £958 6s. 8d. ; Hospital for Consumption, Brompton, £1,458 6s. 8d. ; North London Consumption Hospital, Hampstead, £312 10s. ; Royal Hospital for Diseases of the Chest, City Road, £333 6s. 8d. ; Royal National Hospital for Consumption, Ventnor, £312 10s.

Twelve Children's Hospitals.—Alexandra Hospital for Hip Disease in Childhood, W.C., £208 6s. 8d. ; Belgrave Hospital for Children, S.W., £35 8s. 4d. ; Cheyne Hospital for Incurable Children, S.W., £140 12s. 6d. ; East London Hospital for Children, Shadwell, E., £572 18s. 4d. ; Evelina Hospital for Sick Children, Southwark, S.E., £520 16s. 8d. ; Home for Incurable Children, Maida Vale, W., £72 18s. 4d ; Home for Sick Children, Sydenham, S.E., £145 16s. 8d. ; Hospital for Sick Children, Great Ormond Street, W.C., £791 13s. 4d. ; North-Eastern Hospital for Children, Hackney Road, N.E., £395 16s. 8d. ; Paddington Green Hospital for Children, W., £166 13s. 4d. ; Victoria Hospital for Children, King's Road, Chelsea, S.W., £437 10s. ; "The Vine," Sevenoaks, £33 9s. 2d.

Five Lying-in Hospitals.—British Lying-in Hospital, Endell Street, W.C., £52 1s. 8d. ; City of London Lying-in Hospital, City Road, E.C., £78 2s. 6d. ; Clapham Maternity Hospital, £15 12s. 6d. ; General Lying-in Hospital, Lambeth, S.E., £62 10s. ; Queen Charlotte's Lying-in Hospital, Marylebone Road, W., £312 10s.

Six Hospitals for Women.—Chelsea Hospital for Women, S.W., £208 6s. 8d. ; Hospital for Women, Soho Square, W., £437 10s. ; Grosvenor Hospital for Women and Children, Vincent Square, S.W., £78 2s. 6d. ; New Hospital for Women, Euston Road, W.C., £140 12s. 6d. ; Royal Hospital for Children and Women, Lambeth, S.E., £197 18s. 4d. ; Samaritan Free Hospital, Lower Seymour Street, W., £552 1s. 8d.

#### Twenty-five other Special Hospitals.

Cancer Hospital, Brompton, S.W., £572 18s. 4d. ; London Fever Hospital, Islington, N., £312 10s. ; Gordon Hospital for Fistula, Vauxhall Bridge Road, S.W., £43 17s. 6d. ; St. Mark's Hospital for Fistula, City Road, E.C., £114 11s. 8d. ; National Hospital for the Diseases of the Heart and Paralysis, Soho Square, W., £104 3s. 4d. ; Female Lock Hospital, Harrow Road, W., £302 1s. 8d. ; Male Lock Hospital, Soho, W., £31 5s. ; Hospital for Epilepsy, Paralysis, and other Diseases of the Nervous System, Regent's Park, N.W., £62 10s. ; National Hospital for the Paralysed and Epileptic, W.C., £687 10s. ; Central London Ophthalmic Hospital, Gray's Inn Road, W.C., £43 17s. 6d. ; Royal London Ophthalmic Hospital, Moorfields, E.C., £572 18s. 4d. ; Royal South London Ophthalmic Hospital, St. George's Circus, S.E., £67 14s. 2d. ; Royal Westminster Ophthalmic Hospital, Charing Cross, W.C., £93 15s. ; Western Ophthalmic Hospital, Marylebone Road, W., £13 10s. 10d. ; City Orthopædic Hospital, Hatton Garden, E.C., £62 10s. ; National Orthopædic Hospital, Great Portland Street, W., £46 17s. 6d. ; Royal Orthopædic Hospital, Oxford Street, W., £83 6s. 8d. ; Hospital for Diseases of the Skin, Stamford Street, S.E., £20 16s. 8d. ; St. Peter's Hospital for Stone, Covent Garden, £36 9s. 2d. ; Central London Throat and Ear Hospital, Gray's Inn Road, W.C., £41 13s. 4d. ; Hospital for Diseases of the Throat, Golden Square, W.C., £26 0s. 10d. ; Royal Ear Hospital, Frith Street, W., £5 4s. 2d. ; London Homœopathic Hospital, Great Ormond Street, W.C., £153 5s. ; Dental Hospital of London, Leicester Square, W.C., £104 3s. 4d. ; National Dental Hospital, 149, Great Portland Street, W., £26 0s. 10d.

#### Twenty-one Convalescent Hospitals.

Metropolitan Convalescent Institution, Sackville Street, W., £677 1s. 8d. ; Bexhill Convalescent Home, Sackville Street, W., £333 6s. 8d. ; All Saints' Convalescent Hospital, Eastbourne, £572 18s. 4d. ; Mrs. Gladstone's Convalescent Home, Woodford, £135 8s. 4d. ; Hanwell Convalescent Home, £20 16s. 8d. ; Herbert Convalescent Home, Bournemouth, £41 13s. 4d. ; King's College Hospital Convalescent Home, Hemel Hempstead, £104 3s. 4d. ; Mrs. Kitto's Convalescent Home, Reigate, £83 6s. 8d. ; Mrs. Marshman's London and Brighton Female Convalescent Home, £78 2s. 6d. ; Mary Wardell Convalescent Home for Scarlet Fever, £125 ; Morley House Convalescent Home for Working Men, £52 1s. 8d. ; Princess Frederica's Convalescent Home, East Molesey, £36 9s. 2d. ; Children's Convalescent Home, Ramsgate, £20 16s. 8d. ; St. Andrew's Convalescent Hospital, Clewer, £177 1s. 8d. ; St. Andrew's Convalescent Home, Folkestone, £208 6s. 8d. ; St. Barnabas Convalescent Home, Ramsgate, £20 16s. 8d. ; St. John's Home for Convalescent and Crippled Children, Brighton, £26 0s. 10d. ; Convalescent Home for Poor Children, St. Leonards-on-Sea, £125 ; St. Mary Magdalene's Convalescent Home, Paddington, £52 1s. 8d. ; St. Michael's Convalescent Home, Westgate-on-Sea, £43 17s. 6d. ; Seaside Convalescent Hospital, Seaford, £192 14s. 2d.

#### Fourteen Cottage Hospitals.

Beckenham Cottage Hospital, £46 17s. 6d. ; Blackheath and Charlton Cottage Hospital, £62 10s. ; Bromley, Kent, Cottage Hospital, £57 5s. 10d. ; Burstead Cottage Convalescent Home for Children, £20 16s. 8d. ; Charlwood, Surrey, Cottage Hospital, £5 4s. 2d. ; Chislehurst, Sidcup, and Cray Valley Cottage Hospital, £52 1s. 8d. ; Eltham Cottage Hospital, £20 16s. 8d. ; Enfield Cottage Hospital, £41 13s. 4d. ; Epsom and Ewell Cottage Hospital, £57 5s. 10d. ; Hatfield Broad Oak Cottage Hospital, £5 4s. 2d. ; Reigate and Redhill Cottage Hospital, £72 18s. 4d. ; Shedfield Cottage Hospital and Convalescent Home, £5 4s. 2s. ; Sidcup Cottage Hospital, £31 5s. ; Wimbledon Cottage Hospital, £41 13s. 4d.

#### Nine Institutions.

Establishment for Gentlewomen, Harley Street, £83 6s. 8d. ; Hampstead Home Hospital and Nursing Institute, £88 10s. 10d. ; National Sanatorium for Consumption, Bournemouth, £78 2s. 6d. ; Royal Sea Bathing Infirmary, Margate, £604 3s. 4d. ; Invalid Asylum, Stoke Newington, £72 18s. 4d. ; Firs Home, Bournemouth, £31 5s. ; St. Catherine's Home, Ventnor, £41 13s. 4d ; Hahnemann Convalescent Home, Bournemouth, £20 16s. 8d. ; Royal Mineral Water Hospital, Bath, £41 13s. 4d.

#### Fifty-two Dispensaries.

Battersea Provident Dispensary, £62 10s. ; Brixton, &c., Dispensary, £52 1s. 8d. ; Brompton Provident Dispensary, £26 0s. 10d. ; Camberwell Provident Dispensary, £98 19s. 2d. ; Camden Provident Dispensary, £11 9s. 2d. ; Chelsea, Brompton, and Belgrave Dispensary, £41 13s. 4d. ; Child's Hill Provident Dispensary, £10 8s. 4d. ; City Dispensary, £98 19s. 2d. ; City of London and East London Dispensary, £26 0s. 10d. ; Clapham General and Provident Dispensary, £36 9s. 2d. ; Eastern Dispensary, £31 5s. ; Farringdon General Dispensary, £43 17s. 6d. ; Finsbury Dispensary, £62 10s. ; Forest Hill Dispensary, £36 9s 2d. ; Gipsy Hill and Upper Norwood Dispensary, £15 12s. 6d. ; Hackney Provident Dispensary, £5 4s. 2d. ; Hampstead Provident Dispensary, £46 17s. 6d. ; Holloway and North Islington Dispensary, £78 2s. 6d. ; Islington Dispensary, £46 17s. 6d. ; Kensal Town Provident Dispensary, £13 10s. 10d ; Kensington Dispensary, £72 18s. 4d. ; Kilburn, Maida Vale, and St. John's Wood Dispensary, £46 17s. 6d. ; Kilburn Provident Medical Institution, £31 5s. ; London Dispensary, £20 16s. 8d. ; London Medical Mission, Endell Street, £62 10s. ; Margaret Street Infirmary for Consumption, £12 10s ; Metropolitan Dispensary, £41 13s. 4d. ; Notting Hill Dispensary and Maternity (Provident), £20 16s. 8d. ; Paddington Provident Dispensary, £41 13s. 4d. ; Pimlico Provident Dispensary (Lupus Street), £20 16s. 8d. ; Portland Town Dispensary, £20 16s. 8d. ; Portobello Road Provident Dispensary, £5 4s. 2d ; Public Dispensary, £52 1s. 8d. ; Queen Adelaide's Dispensary, £41 13s. 4d. ; Royal General Dispensary, £41 13s. 4d. ; Royal Pimlico Provident Dispensary, £46 17s. 6d. ; Royal South London Dispensary, £67 14s. 2d. ; St. George's and St. James's Dispensary, £57 5s. 10d. ; St. George's, Hanover Square, Provident Dispensary, £52 1s. 8d. ; St. John's Wood and Portland Town Provident Dispensary, £26 0s. 10d. ; St. Marylebone General Dispensary, £36 9s. 2d. ; St. Pancras and Northern Dispensary, £43 17s. 6d. ; South Lambeth, Stockwell, and North Brixton Dispensary, £57 5s. 10d. ; South London Medical Aid Institute, £20 16s. 8d. ; Stamford Hill, Stoke Newington, &c., Dispensary, £43 17s. 6d. ; Tower Hamlets Dispensary, £46 17s. 6d. ; Wandsworth Common Provident Dispensary, £10 8s. 4d. ; Westbourne Provident Dispensary and Maternity, £20 16s. 8d. ; Western Dispensary, £46 17s. 6d. ; Western General Dispensary, £145 13s. 8d. ; West Ham, Stratford, and South Essex Dispensary, £93 15s. ; Westminster General Dispensary, £31 5s.

One hundred and eighty-eight pounds in all will be deducted from certain of the above sums, being the amounts received from collections sent direct to particular institutions,

